# Giant ovarian cyst presenting with reversible cardiac compromise: a case report

**DOI:** 10.1097/RC9.0000000000000212

**Published:** 2026-02-06

**Authors:** Diwakar Koirala, Bivek Mishra, Yamuna Agrawal, Yogesh Dhakal, Ramesh Shrestha

**Affiliations:** a Department of Obstetrics and Gynecology, B. P. Koirala Institute of Health Sciences, Dharan, Nepal; b Department of Pathology, B. P. Koirala Institute of Health Sciences, Dharan, Nepal; c Department of Anesthesiology and Critical Care, B. P. Koirala Institute of Health Sciences, Dharan, Nepal; d Department of Obstetrics and Gynaecology, B.P. Koirala Institute of Health Sciences, Dharan, Nepal

**Keywords:** borderline mucinous tumor, cardiac compromise, giant ovarian cyst, multidisciplinary approach, surgery

## Abstract

**Introduction and importance:**

Giant ovarian cysts are rare in contemporary practice due to early radiological detection. Cardiac compromise resulting from mechanical compression by such masses is exceptionally uncommon and poses significant diagnostic and perioperative challenges, particularly in resource-limited settings.

**Case presentation:**

A 39-year-old woman presented with a 4-month history of progressive abdominal distension, fatigue, and dyspnea. Imaging revealed a massive multiloculated cystic ovarian mass measuring 38 × 31 × 24 cm, compressing surrounding viscera. Transthoracic echocardiography demonstrated global left ventricular hypokinesia with a reduced ejection fraction of 30–35%, consistent with cardiac dysfunction secondary to mass effect. The patient underwent fertility-preserving staging laparotomy, including right salphingo-oophorectomy. The excised mass measured 40 × 40 cm and weighed 18.6 kg. Histopathology confirmed a borderline mucinous cystadenoma confined to the right ovary (FIGO stage IA).

**Clinical discussion:**

Giant ovarian cysts can rarely induce reversible cardiac dysfunction through elevated intra-abdominal pressure and impaired venous return. Early echocardiographic assessment and multidisciplinary coordination are essential for risk stratification and perioperative planning. In this case, cardiac function improved following tumor removal, supporting a compression-related mechanism rather than intrinsic cardiomyopathy.

**Conclusion:**

This case highlights a rare but life-threatening presentation of reversible cardiac compromise caused by a giant ovarian cyst. Timely diagnosis, echocardiographic evaluation, and coordinated surgical management enabled a favorable outcome despite limited resources, emphasizing the importance of early intervention in similar high-risk presentations.

## Introduction

Giant ovarian cysts, typically defined as measuring over 10–15 cm in diameter, are exceptionally uncommon in younger individuals^[1]^. Their rarity today is largely due to early incidental detection through modern radiological imaging. Management depends on tumor size, patient age, and histological type^[2]^. Reported prevalence ranges from 37% to 66% in perimenopausal women and 18–86% in postmenopausal women^[3]^. Patients usually present with abdominal pain, distention, or pressure-related symptoms, though misdiagnosis as pancreatic, hydatid, or mesenteric cysts may occur. While most giant ovarian cysts are benign, a subset may be malignant or display borderline histology^[4]^.

**HIGHLIGHTS**

- Giant ovarian cyst caused reversible cardiac compromise due to compression.
- Multidisciplinary surgical care led to full cardiac and clinical recovery.
- Histopathology confirmed borderline mucinous cystadenoma of right ovary.
- Early imaging and echocardiography were crucial for timely diagnosis.
- Fertility-sparing surgery was successful even in a resource-limited setting.

Beyond abdominal manifestations, giant cysts can occasionally produce systemic complications. Mechanical compression of the diaphragm, great vessels, and adjacent organs may impair venous return and cardiac filling, precipitating hemodynamic instability or overt heart failure. Although rare, such cardiac compromise can manifest as reduced ejection fraction, arrhythmias, or right-sided heart failure, posing a significant perioperative risk. Early echocardiographic assessment is therefore essential to identify and manage these high-risk presentations.

Diagnosis is generally straightforward with ultrasonography, although CT or MRI can be required for characterization, and serological tests including *Echinococcus* titers and tumor markers (CA-125, CA-19-9, CEA) may aid evaluation^[5]^. Traditionally managed with laparotomy, giant ovarian cysts are increasingly approached laparoscopically in specialized centers^[6]^.

The present case report highlights the rare occurrence of cardiac compromise secondary to a giant ovarian cyst, underscoring the importance of early imaging, multidisciplinary collaboration, and pragmatic surgical strategies in resource-limited settings.

The report has been written in accordance with the SCARE criteria^[7]^.

## Case report

A 39-year-old female, nulliparous presented with a 4-month history of progressive abdominal distension and difficulty in walking. Initially, she noted mild abdominal bloating, which she attributed to dietary changes. Over time, the bloating worsened, accompanied by vague abdominal discomfort and a sensation of heaviness. She began experiencing fatigue and reduced appetite, leading to unintentional weight loss. The patient also reported occasional episodes of nausea but denied vomiting, fever, or changes in bowel habits. Over the past month, she developed increasing difficulty in walking due to abdominal discomfort and weakness. Seeking care at multiple facilities, she was advised surgical intervention. However, financial constraints delayed definitive management.

There was no history of:

- hormonal therapy;
- family history of malignancies, including ovarian or breast cancer; and
- prior abdominal surgeries

**Figure 1:**
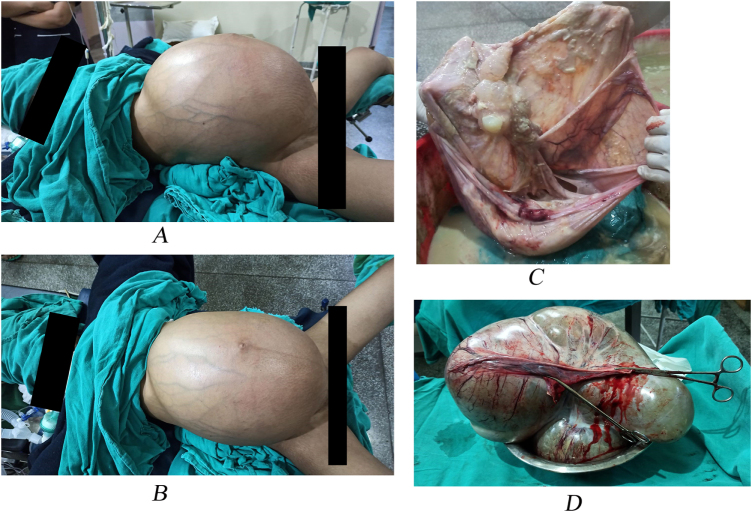
(A, B): Preoperative clinical photographs demonstrating marked abdominal distension due to the giant ovarian cyst. (C, D): Postoperative images of the excised mass.

On examination, the patient appeared cachexic with ECOG performance status of 2. Her body mass index was 19.4 kg/m^2^. Vital signs were within normal limits. Breast and thyroid were normal with no palpable peripheral lymph nodes. Abdominal palpation revealed a tense smooth mass extending from pubic symphysis to xiphisternum with mild tenderness and markedly engorged vessels running below the skin. No evidence of ascites was noted clinically. Pelvic examination revealed the same tense cystic mass, but vulva, vagina, and cervix were normal. The examination findings has been mentioned in Fig. 1. The timeline of the events have been summarized in Table 1.

**Table 1:** Timeline of events (POD post-operative day)

Date	Timeline	Event
May 2024	4 months before presentation	Gradual onset of abdominal distension and bloating
August 2024	1 month before presentation	Progressive abdominal discomfort, fatigue, reduced appetite
September 20, 2024	Presentation in the gynecological out-patient clinic, BPKIHS	Worsening abdominal distension, dyspnea on exertion, difficulty in walking
October 8, 2024	Day of Surgery	Fertility-preserving staging laparotomy; removal of 40 × 40 cm ovarian mass (18.6 kg)
October 9, 2024 (POD 1)	Immediate postoperative period	Uneventful recovery; single intraoperative blood transfusion
October 17, 2024 (POD 9)	Postoperative echocardiography and discharge	Improvement in cardiac function (LVEF 40–45%, no pericardial effusion); wound healthy. Ambulating and breathing well and no feeding problems
August 2025	Follow-up (at 10 months postsurgery)	Asymptomatic; normal tumor markers and unremarkable imaging

**Figure 2.**
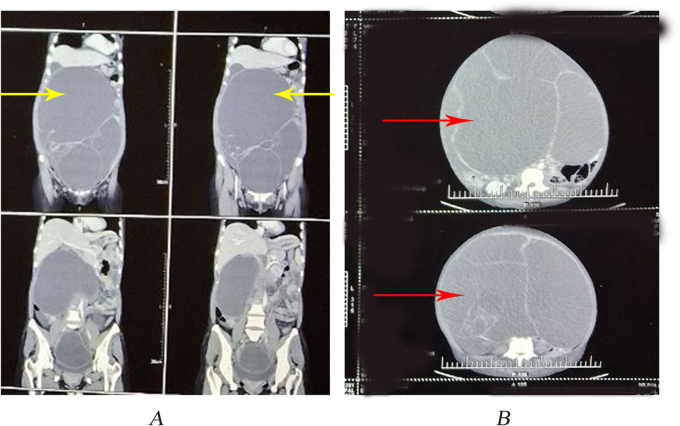
Contrast-enhanced CT of the abdomen and pelvis. (A) Coronal view showing a giant multiloculated cystic abdominopelvic mass (yellow arrows). (B) Axial view demonstrating internal septations and peripheral solid components (red arrows), with compression of surrounding abdominal organs.

Contrast-enhanced computed tomography (CECT) whole abdomen and pelvis revealed a well-defined large multiloculated thick-walled abdominopelvic predominantly cystic mass of size 38 × 31 × 24 cm (CCxTRxAP) with thin and thick enhancing internal septations and peripheral solid component seen occupying the entire abdomen and pelvis with thinning of abdominal wall muscles; superiorly abutting and displacing small bowel and large bowel loops, gall bladder and right lobe of liver; posteriorly abutting abdominal aorta with its branches along with compressing small bowel loops, including ampullary region, pancreas with dilatation of main pancreatic duct (6.4 mm), common bile duct (10 mm) with bilobar IHBRD with transient hepatic arterial attenuation difference in adjacent liver segments. The mass is also compressing bilateral distal ureters with proximal mild dilatation of pelvi-calyceal system of bilateral kidneys. Mild fluid in pelvis. No evidence of enlarged intra-abdominal lymph nodes. Rest normal. B/L ovaries not visualized separately. Likely malignant cystic ovarian neoplasm possibly mucinous carcinoma of ovary. The image findings have been mentioned in Fig. 2. Chest X-ray showed normal lung fields but with hugely pushed hemidiaphragm.

Preoperative echocardiography revealed global left ventricular (LV) wall hypokinesia with ejection fraction (EF) of 30–35%. Minimal pericardial effusion.

Tumor markers analysis showed CA-125: 122 U/ml, CEA: 1.68 ng/ml, CA19-9: 9.86 U/ml, AFP: 4.25 ng/ml, b-HCG: 0.321 mIU/ml, and lactate dehydrogenase (LDH): 295 U/L.

The patient was taken for fertility-preserving-staging laparotomy (peritoneal washings for cytology, right salphingo-ophorectomy with left ovarian biopsy, infracolic omentectomy, pelvic lymphadenectomy, and paraaortic lymphadenectomy) on October 8, 2024 with the provisional diagnosis of ovarian malignancy after informed written consent. Fertility-preserving surgery was offered despite the patient’s age of 39 years, as she expressed a strong desire to preserve fertility potential, and intraoperative findings suggested ovarian confined disease (limited involvement of the contralateral ovary with suspicious surface deposit but no gross invasion). Intraoperative frozen section was not performed due to resource limitations in the setting. The procedure was performed under general anesthesia with epidural analgesia with standard perioperative monitoring. Prophylactic antibiotics (intravenous cefuroxime 30 minutes before skin incision) and venous thromboembolism prophylaxis (mechanical prophylaxis with TED stockings and medical prophylaxis with subcutaneous low molecular weight heparin: one dose 12 hours before surgery and continued 24 hours after surgery given each day till hospital stay) were administered according to institutional protocol. A midline supra-umbilical vertical incision was preferred to allow safe exposure and controlled removal of the mass without rupture. The surgery was performed by an experienced gynecologic oncology team in a tertiary-care public hospital setting. Hemostasis was achieved using standard suturing techniques, and the abdominal wall was closed in layers.

Intraoperative findings revealed minimal clear pelvic ascites. Right ovarian mass measuring 40 × 40 cm (weighing 18.6 kg), bosselated, cystic consistency, capsule intact, no surface deposits. Cut section: multiloculated cyst containing thick cheesy/fatty content with peripheral solid component at one place. Suspicious deposit seen on the surface of left ovary. Appendix normal. Rest of the abdomen (omentum, peritoneum, diaphragms, and viscera) was normal. Intraoperative PCI: 0, SCS = 3, CC = 0.

The surgical duration was 3 hours and received one unit of blood transfusion in the intraoperative period. She did well in the postoperative period and was discharged on 9th postoperative day with no any postoperative complications.

Postoperative echocardiography showed normal chamber dimension and valves. LVEF = 40–45% with no pericardial effusion.

The final histopathological report confirmed the diagnosis of borderline mucinous cystadenoma of the right ovary. Biopsy of the left ovary showed no evidence of malignancy or borderline features. The final stage was FIGO IA, based on the ovarian confined disease on comprehensive staging with no contralateral or extra-ovarian disease/implants and negative peritoneal fluid analysis.

**Figure 3.**
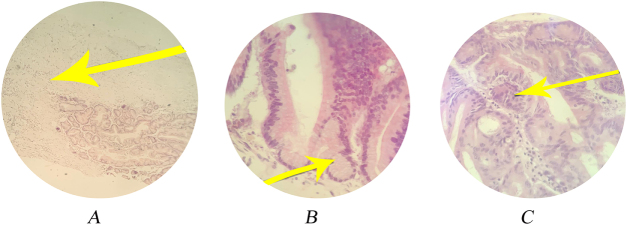
Histopathological features of borderline mucinous cystadenoma. (A) Glandular cystic structures lined by mucinous epithelium. (B) Glandular structures with papillary infoldings consistent with borderline features. (C) Stromal components without evidence of invasive malignancy (hematoxylin and eosin stain)..

The patient was followed up for a total duration of 10 months postsurgery. During the post-treatment surveillance, she remained clinically asymptomatic with no clinical, biochemical, or radiological recurrence The histopathological findings have been mentioned in Fig. 3. Tumor markers, including CA-125, were within normal limits during follow-up. Final histopathological report is given as follows.

## Discussion

Giant ovarian cysts can rarely result in clinically significant cardiac compromise due to sustained mechanical compression of the diaphragm and major abdominal vessels. In the present case, markedly elevated intra-abdominal pressure led to impaired venous return and reduced cardiac output, manifesting as global left ventricular hypokinesia and a reduced ejection fraction. Importantly, cardiac dysfunction improved following surgical excision, supporting a reversible, compression-induced mechanism rather than primary cardiomyopathy^[8]^.

Borderline ovarian tumors (BOTs) remain a diagnostic challenge for pathologists^[9]^. Despite their uncertain malignant potential, they generally carry an excellent prognosis^[10]^ and rarely, manifest as large abdominal cystic masses. A thorough workup should include ultrasound or CT imaging and tumor markers such as CA-125, CA 19-9, and CEA. In adolescents, germ cell markers (β-HCG, LDH, AFP) must also be evaluated. Intraoperatively, careful inspection of the appendix, bowel, and peritoneum is crucial to exclude implants or secondary mucinous neoplasms.

In the past few years, management strategies for BOTs have evolved. Most are detected at an early stage, with 5-year survival rates approaching 95%^[11]^. Traditional treatment involved total abdominal hysterectomy with bilateral salphingo-oophorectomy^[12]^. Today, fertility-preserving surgery is widely practiced in younger patients without compromising oncologic outcomes^[12]^. Unilateral cystectomy, though associated with higher recurrence, does not reduce overall survival^[13]^. Frozen section evaluation can aid intraoperative decision-making, although its sensitivity for BOTs remains limited^[14]^.

Cardiac dysfunction secondary to ovarian tumors has been infrequently reported in the literature. Prior cases have described heart failure associated with ovarian steroid cell tumors^[15]^ and carcinoid tumors^[16]^, where cardiac involvement was mediated by hormonal or vasoactive substances and resolved following tumor resection. In contrast, the cardiac compromise in our case resulted purely from mechanical compression, highlighting a distinct and reversible pathophysiological mechanism.

## Conclusion

This case highlights a rare but serious complication of reduced cardiac function caused by a giant ovarian cyst through elevated intra-abdominal pressure and impaired venous return supporting a compression-related mechanism rather than intrinsic cardiomyopathy. Early recognition with imaging and echocardiography, supported by multidisciplinary care, enabled successful surgical management and their potential to precipitate life-threatening cardiac effects underscores the importance of timely intervention. fertility-preserving strategies can be individualized, even in older reproductive-age women with minimal contralateral disease. Ensuring timely access to care is essential to mitigate disparities and optimize outcomes.

## Research registration unique identifying number (UIN)

Not applicable for case reports.

## Patient perspective

Before the surgery, the patient was extremely weak, short of breath, and the abdominal swelling made daily activities very difficult. The patient was let to shop three tertiary hospitals for the surgical treatment due to financial issues until she was presented to BPKIHS, Dharan. After the operation, the patient gradually felt the strength return, and breathing improved significantly. The patient along with her husband are grateful to the surgical teams, the Department and BPKIHS, Dharan for the timely treatment and follow-up care that helped the patient return to a normal life despite of financial constraints.
